# The pain target Na_V_1.7 is expressed late during human iPS cell differentiation into sensory neurons as determined in high-resolution imaging

**DOI:** 10.1007/s00424-024-02945-w

**Published:** 2024-03-27

**Authors:** Yi Liu, Rachna Balaji, Marcelo A. Szymanski de Toledo, Sabrina Ernst, Petra Hautvast, Aylin B. Kesdoğan, Jannis Körner, Martin Zenke, Anika Neureiter, Angelika Lampert

**Affiliations:** 1 Institute of Neurophysiology, Uniklinik RWTH Aachen, Pauwelsstrasse 30, 52074 Aachen, Germany; 2 Department of Hematology, Oncology, and Stem Cell Transplantation, Uniklinik RWTH Aachen, Pauwelsstrasse 30, 52074 Aachen, Germany; 3 Confocal Microscopy Facility, Interdisciplinary Center for Clinical Research IZKF, Uniklinik RWTH Aachen, Pauwelsstrasse 30, 52074 Aachen, Germany; 4 Department of Anaesthesiology, Uniklinik RWTH Aachen, Pauwelsstrasse 30, 52074 Aachen, Germany; 5 Department of Intensive and Intermediate Care, Uniklinik RWTH Aachen, Pauwelsstrasse 30, 52074 Aachen, Germany; 6 Scientific Center for Neuropathic Pain Research Aachen, SCN-Aachen Uniklinik RWTH Aachen, Aachen, Germany

**Keywords:** HA-tag, Stem cell–derived sensory neurons, Membrane localization, CRISPR-Cas, Disease modeling

## Abstract

Human-induced pluripotent stem cells (iPS cells) are efficiently differentiated into sensory neurons. These cells express the voltage-gated sodium channel Na_V_1.7, which is a validated pain target. Na_V_1.7 deficiency leads to pain insensitivity, whereas Na_V_1.7 gain-of-function mutants are associated with chronic pain. During differentiation, the sensory neurons start spontaneous action potential firing around day 22, with increasing firing rate until day 40. Here, we used CRISPR/Cas9 genome editing to generate a HA-tag Na_V_1.7 to follow its expression during differentiation. We used two protocols to generate sensory neurons: the classical small molecule approach and a directed differentiation methodology and assessed surface Na_V_1.7 expression by Airyscan high-resolution microscopy. Our results show that maturation of at least 49 days is necessary to observe robust Na_V_1.7 surface expression in both protocols. Electric activity of the sensory neurons precedes Na_V_1.7 surface expression. A clinically effective Na_V_1.7 blocker is still missing, and we expect this iPS cell model system to be useful for drug discovery and disease modeling.

**Supplementary Information:**

The online version contains supplementary material available at 10.1007/s00424-024-02945-w.

## Introduction

The voltage‐gated sodium channel Na_V_1.7, encoded by the gene *SCN9A* in humans, is expressed peripherally in dorsal root ganglion neurons (DRGs), trigeminal ganglia, and sympathetic neurons [2], as well as in the central nervous system, albeit at lower expression levels [3, 15]. Na_V_1.7 as a threshold channel plays a key role in initiating action potentials by generating fast‐activating and fast‐inactivating currents, which contribute to the generation and propagation of action potentials [19]. Gain-of-function mutations in Na_V_1.7 cause human pain disorders, such as inherited erythromelalgia (IEM) [16, 18] and small fiber neuropathy (SFN) [10], whereas loss-of-function mutations of Na_V_1.7 lead to congenital insensitivity to pain (CIP) [7, 13]. An enormous effort has been made for several decades to develop novel analgesics by selectively targeting Na_V_1.7. Unfortunately, so far, many of these blockers have failed to show efficacy in clinical trials [9].

One reason for the slow development of new effective drugs targeting Na_V_1.7 may reside in the lack of translational potential of basic research results into clinics. Human embryonic kidney 293 (HEK293) cell lines are used for studying the biophysics and pharmacology of ion channels, such as Na_V_1.7, but their cellular background is not neuronal, and associated proteins or essential modifying factors may be missing [12, 32]. Thus, the gating behavior in HEK cells may not reflect the channel’s function in a more physiological context [19, 29]. Animal models are highly useful to determine systemic effects and understand complex mechanisms; nevertheless, expression of pain-related genes differs between rodent and humans [28, 39]. Thus, a human sensory neuronal model is critically needed.

The reprogramming of adult somatic cells into human-induced pluripotent stem cells (hiPSCs) and their differentiation into any cell type has revolutionized the study of human-specific cells for mechanistic and pre-clinical studies [33, 34]. It has enabled studying molecular mechanisms and signaling pathways in vitro from both healthy individuals and patients, offsetting the limited access to human DRG or other neuronal tissues. hiPSCs recapitulate the genetic background of the donor throughout the differentiation procedures [17]. The versatility of patient-derived hiPSCs, their effective differentiation towards functional nociceptor-like neurons [5] from various patients with and without known genetic alteration, and the ability for clustered regularly interspaced short palindromic repeats (CRISPR)-Cas9 gene editing have provided a technology platform for understanding disease pathophysiology.

The use of iPSC-derived sensory neurons (iPSC-SNs) from pain patients has been shown to be useful to study Na_V_1.7 and human nociception. Several protocols are currently used, whereas the most commonly applied protocol uses small molecules [5]. Electrophysiological assessment of iPSC-SNs generated using this protocol from IEM or SFN patients showed increased spontaneous activity and hyper-excitability in response to increasing temperatures [4, 19, 21, 23]. Similar studies revealed that iPSC-SNs exhibit reduced nociceptor excitability to depolarizing stimuli from CIP patients [18]. As the Chambers protocol is time intensive and reveals a mixture of different cell types (although potentially similar to what is observed in native sensory neuron ganglia), efforts to produce a better-defined neuronal population within a shorter time were undertaken. One of the most promising protocols uses virus-mediated forced expression of NGN-1 [30].

Na_V_1.7 needs to be expressed on the cell surface to fulfill its function. However, a previous study showed that Na_V_1.7 is functionally expressed in iPSC-SNs derived by the Chambers protocol only at day 65 of differentiation, although mRNA was detected already at day 30 [19]. Therefore, precise tracking of surface localization of Na_V_1.7 protein is essential to be able to study and understand the functional role of Na_V_1.7 in the human nociceptive system.

However, the poor specificity of commercially available Na_V_1.7 antibodies for immunostaining hampers the efforts to determine subcellular protein localization. Epitope tagging is a widely used technique in which a protein of interest is fused to a sampler tag by genetic engineering [22]. One widely used epitope tag is hemagglutinin (HA), which has a nine amino acid sequence (YPYDVPDYA) from the human influenza virus hemagglutinin protein [11, 36], for which reliable antibodies are commercially available. Labeling of endogenous proteins with HA-tag using CRISPR-Cas9 has been accomplished in mammalian brain tissues in vivo [20], as well as in iPSC-SNs in vitro [18]. The latter study showed evidence for surface expression of HA-tagged Na_V_1.7 around day 60 of small molecule differentiation [5], while at day 25, the HA signal was localized mainly intracellularly [18]. The continuous changes of Na_V_1.7 expression during several time points of this differentiation in comparison to the NGN-1 forced expression protocol and the exact starting point of surface localization of Na_V_1.7 are still unknown.

To fill this gap, we used hiPSCs and fused the gene *SCN9A* C-terminally with an HA-tag that allows reliable immunofluorescence detection of Na_V_1.7 during the time course of differentiation. We compare two differentiation procedures to derive sensory neurons from the HA-tagged hiPSCs: (1) the widely used small molecule–based differentiation procedure first published by Chambers and colleagues [5] (referred to as small molecule protocol) with some modifications and (2) a recently published method relying on forced expression of NEUROGENIN1 (NGN1) in neural crest–like cells (NCLCs) (referred to as NGN1 protocol) [30]. Both protocols result in neurons expressing nociceptor-associated genes, including *SCN9A* on mRNA level. Using high-resolution confocal microscopy, we tracked Na_V_1.7 protein expression and cellular localization during differentiation to pin down the time point of clear cell surface localization of the protein, a prerequisite for the use of iPSC-derived sensory neurons for in vitro drug development and substance testing in the future.

## Results

### Morphological comparison of small molecule and forced expression iPSC-SN protocols

**Fig. 1:**
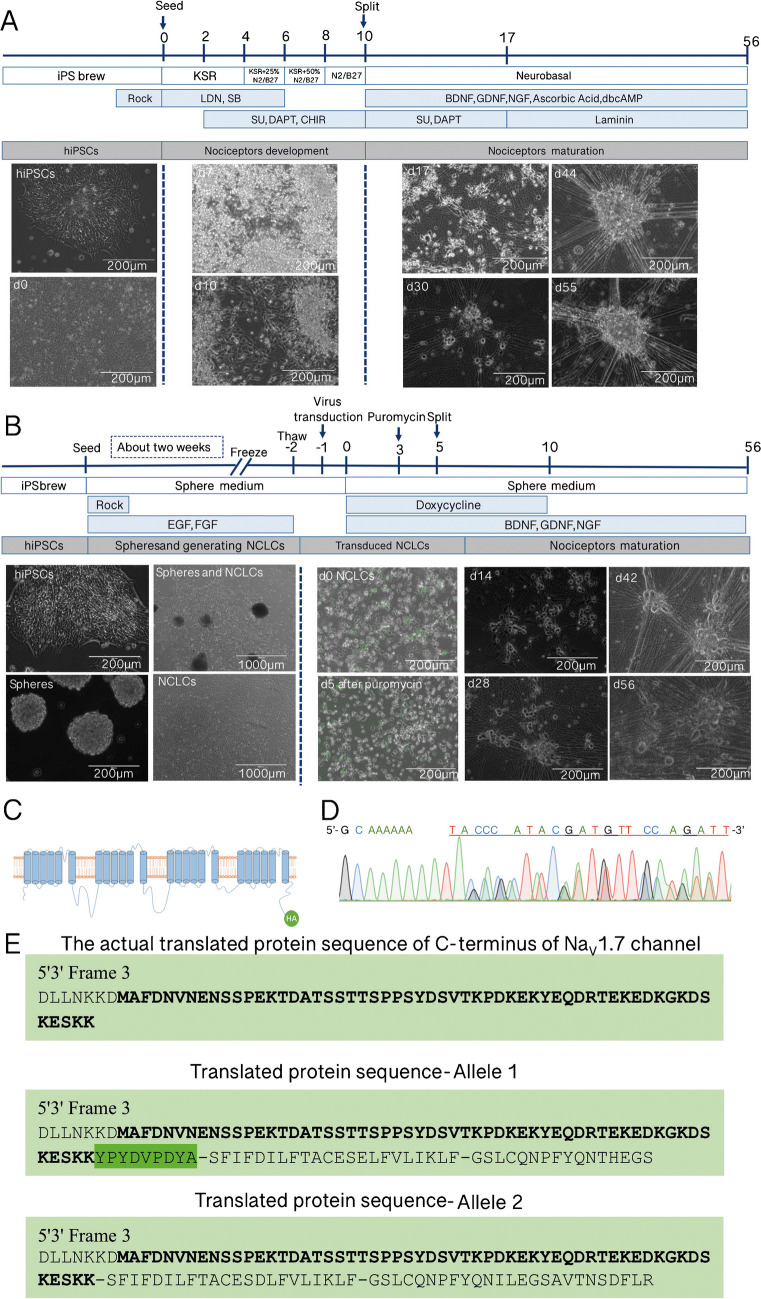
iPSC-SNs were generated, and the monoallelic HA-tag by CRISPR-Cas9-mediated genome editing was detectable. **A** Schematic illustration of small molecule differentiation protocol to generate iPSC-SNs from hiPSCs: horizontal line indicates time points of iPSC-SNs development and maturation. KSR, medium supplemented with knockout serum replacement; N2/B27, medium supplemented with N2, B27 (scale bar 200 µm). **B** Schematic illustration of NGN1 differentiation protocol to generate iPSC-SNs from hiPSCs: horizontal line indicates time points of NCLC generation, virus infection, selection (addition of puromycin), and splitting of cells. Pictures show characteristic morphology of cells at the indicated time points: d0 neural crest–like cells (NCLCs), d5 NGN1 expression, indicated by GFP signal; d14 neural progenitors show small neurites; ≥ 28 d neurons with long neurites can be observed. × 4 magnification: scale bar 1000 µm, × 20 magnification: scale bar 200 µm. **C** A HA epitope tag, indicated in green, present at the C-terminus of the Na_V_1.7 channel using CRISPR-Cas9-mediated genome editing. **D** Overlaying sequences of Sanger sequencing by CRISP-ID: the HA-tag sequence TACCCATACGATGTTCCAGATT was present in the chosen subclone. **E** Sequencing results showing the presence of a monoallelic HA-tag. Translated protein sequences (5′ to 3′) of the C-terminus of Na_V_1.7 channel of both the alleles of Na_V_1.7 HA-tagged hiPSC_113.16 subclone. The open reading frame of the Na_V_1.7 channel protein sequence is highlighted in bold; HA-tag protein sequence (YPYDVPDYA) is highlighted in green

In our experiments, we compared two widely used nociceptor differentiation protocols to generate iPSC-SNs [5, 24, 30]: a small molecule–based differentiation protocol with some modifications as shown in Fig. 1A and a forced expression protocol in Fig. 1B.

For the small molecule approach, the cells grow in a dense monolayer at day 0 and exhibit a typical hiPSCs morphology with a large nucleus and less cytoplasm. Subjected to the defined signaling pathway modulation by small molecules, cells started exhibiting neuron-like morphology with a clear cell body and neurites growing out from d7 to d10. Thus, at this time point, the cells show obvious neuronal morphology, and at d17, d30, and d44 of differentiation, the formation of sensory neuron ganglion–like structures together with presence of proliferative non-neuronal cells was evident. These sensory ganglion–like structures become more mature and larger in size until d55 (Fig. 1A).

The forced expression method starts with the formation of spheres from hiPSCs as depicted in Fig. 1B. The spheres grew in size, and after 10–14 days of sphere culture, they attach and neural crest–like cells (NCLCs) migrate out of the spheres. By transducing the NCLCs with lentiviruses carrying NGN1 and a puromycin-resistance cassette, the differentiation of sensory neurons is induced by addition of doxycycline (= d0, see the “Methods” section). At d5 to d14, immature neurons with small neurites appear. From d28 of differentiation, the formation and the growth of sensory neuron ganglion–like structures is clearly evident. iPSC-SNs become more mature with enlarged cell size at d42 and d56 compared to d28 (Fig. 1B).

### Electric neuronal activity during small molecule differentiation

**Fig. 2:**
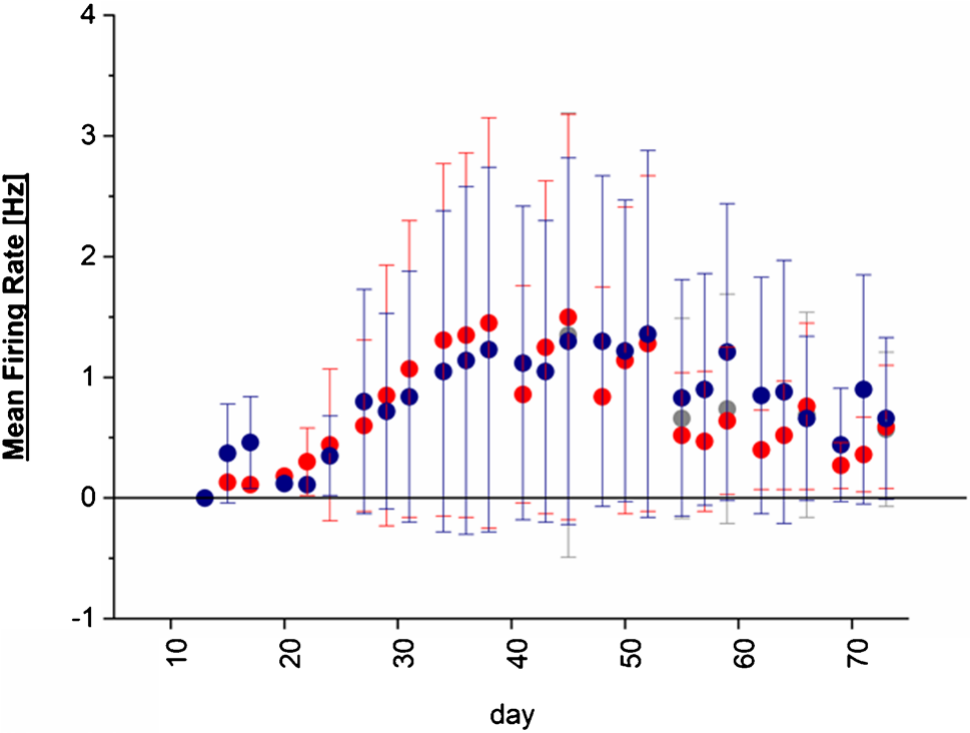
Mean firing rate [Hz] of sensory neurons differentiated by small molecule–driven differentiation. The non-HA-tagged iPSCs were differentiated to sensory neurons, and the development of the mean firing rate was tracked over the time course of differentiation by high-density multi-electrode array recordings. The mean firing rate [Hz] + / − SD per well of three independent differentiations (color coded: red, blue, gray) is shown

To assess at which time point during the differentiation the iPSC-SNs start to generate action potentials, we used multi-electrode arrays and monitored the development of the electric activity during three differentiations of iPSCs into sensory neurons (Fig. 2). We detect spontaneous action potential firing starting at day 22 by an increased mean firing rate, which continuously increases until day 40 of differentiation. Since sodium channels have a key role in the generation of action potentials, these data show that sodium channels start to be expressed from day 22 onwards. Thus, we hypothesize that Na_V_1.7 may be part of those sodium channel subtypes which start to be upregulated with the onset of electric activity. As specific antibodies for Na_V_1.7 are scarce, we decided to genetically engineer the iPSC cell line used for the multi-electrode array recordings in which we fuse an HA-tag to one allele of the SCN9a gene.

### Generation of HA-tagged Na_V_1.7 hiPSC line

The HA-tagged Na_V_1.7 hiPSC line was generated to monitor the expression of Na_V_1.7 along the time course of nociceptor differentiation. For that, the HA-tag coding sequence was inserted into the C-terminus of *SCN9A* gene (Fig. 1C). Successful insertion of the HA-tag was confirmed by polymerase chain reaction (PCR) (Fig. S1A-C). To confirm the presence of a monoallelic tag causing the overlap, the HA-tag sequence was identified by using CRISP-ID, a web application developed by the researchers from KU Leuven [8] that uses a unique algorithm for the genotyping of up to three alleles from a Sanger sequencing trace (Fig. 1D). The presence of the HA-tag nucleotide sequence in the 3′ end of one of the alleles of *SCN9A* gene was confirmed. To exclude that the overlapping sequences result from different cells/clones, one carrying the tag and the other one being untagged, we performed an additional clonal expansion and repeated the sequencing with the resulting clones (Fig.S1B-C). The clones confirmed the presence of a monoallelically tagging of the *SCN9A* gene. In silico analysis showed the translated protein sequences of the C-terminus of the Na_V_1.7 channel of both the alleles from one of HA-tagged Na_V_1.7 hiPSC_113 subclones (Fig. 1E), which further demonstrates the presence of the HA-tag protein sequence (YPYDVPDYA) at the C-terminus of one of the alleles of Na_V_1.7 (Sequence 1) but without any insertion (Sequence 2). These results indicate the successful generation of HA-tagged Na_V_1.7 hiPSC lines. The chosen cell lines HA-tagged Na_V_1.7 hiPSC_113 subclones which have been applied for further analysis maintain the expression of the pluripotency-associated markers OCT4A, SOX2, and NANOG (Fig.S1D). At the same time, 97% of the cells express TRA1-60 and SSEA4 which indicate their pluripotency state (Fig.S1E).

### mRNA expression of nociceptive genes occurs at distinct time points for the compared differentiation methods

**Fig. 3:**
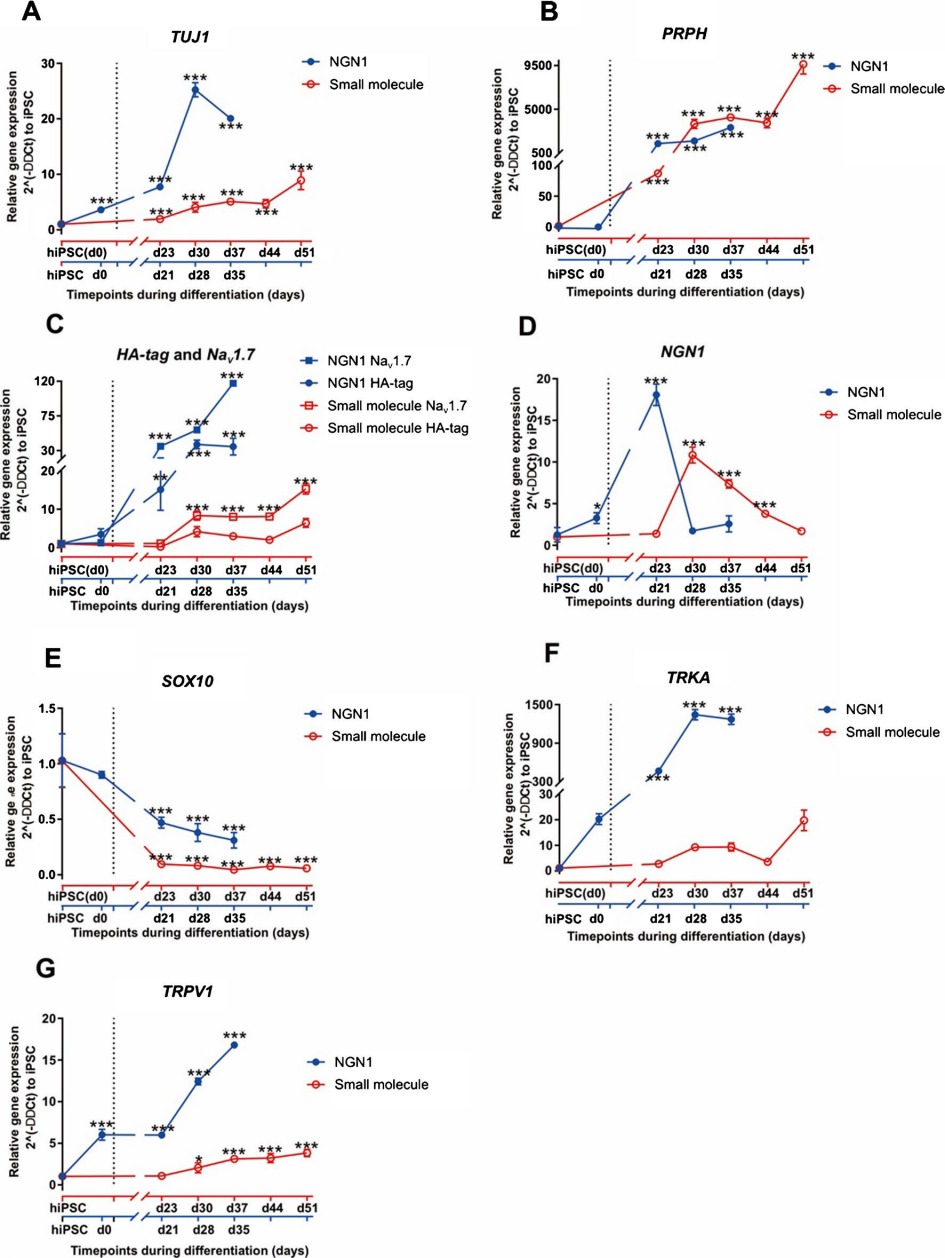
Neuronal marker gene expression changes during both iPSC-SNs differentiation. **A**, **B** qRT-PCR analysis of *TUJ1* and *PRPH*, in iPSC-SNs at different time points during their differentiation (NGN1 differentiation (blue): NCLCs, d21, d28, and d35; small molecule differentiation (red): hiPSC, d23, d30, d37, d44, and d51; relative to the housekeeper *GAPDH* and normalized to hiPSC). Blue square refers to gene expressions of NGN1 differentiation; red circle refers to gene expressions of small molecule differentiation (*n* = 3 independent replicates). **C** qRT-PCR analysis of *Na*_V_*1.7* and *HA-tag* in iPSC-SNs at different time points during their differentiation (NGN1 differentiation: NCLCs, d21, d28, and d35; small molecule differentiation: hiPSC, d23, d30, d37, d44, and d51, relative to the housekeeper *GAPDH* and normalized to hiPSC). Blue square refers to *Na*_V_*1.7* mRNA expression of NGN1 differentiation; blue circle refers to *HA-tag* expression of NGN1 differentiation; red square refers to *Na*_V_*1.7* mRNA expression of NGN1 differentiation; red circle refers to *HA-tag* expression of NGN1 differentiation (*n* = 3 independent replicates). **D**–**G** qRT-PCR analysis of *NGN1*, *SOX10*, *TRKA*, and *TRPV1* in iPSC-SNs at different time points during their differentiation (NGN1 differentiation: NCLCs, d21, d28, and d35; small molecule differentiation: hiPSC, d23, d30, d37, d44, and d51; relative to the housekeeper *GAPDH* and normalized to hiPSC). Blue square refers to gene expressions of NGN1 differentiation; red circle refers to gene expressions of small molecule differentiation (*n* = 3 independent replicates)

We surveyed the expressions of several nociceptor-associated genes on mRNA level via quantitative real-time PCR (qRT-PCR) and found a significant increase of mRNA expression of a neuron-specific class III beta-*tubulin* (*TUJ1*) and peripheral cytoskeletal gene *peripherin* (*PRPH*) throughout the differentiation relative to the level of iPSCs, indicating the generation of neurons of the peripheral nervous system. There is a burst increase in the mRNA expression of* TUJ1* around d30 of NGN1 differentiation, and the expression of *TUJ1* is continuously increasing from d23 to d51 of small molecule differentiation (Fig. 3A). There is also a burst increase in mRNA expression of *PRPH* from d23 of small molecule differentiation and d21 of NGN1 differentiation (Fig. 3B). Apart from these two peripheral neural markers, we monitored the mRNA expression of *HA-tag* and *Na*_V_*1.7*. We found the upregulation of *HA-tag* was changing in parallel to the increasing expression of *Na*_V_*1.7* in both protocols (Fig. 3C). The expression of *Na*_V_*1.7* mRNA already started around d20 and kept increasing following the time course of differentiation. As expected for a haplotypic cell line, the *HA-tag* expression is approximately 50% less than that of *Na*_V_*1.7* expression at all time points during the course of small molecule differentiation. This confirms the monoallelic expression of the *HA-tag* which also correlates with the sequencing results (Fig. 3C).

The upregulation of *NGN1* during the time course of nociceptor’s maturation indicated the presence of nociceptors in both differentiation approaches (Fig. 3D). The highest upregulation of *NGN1* appeared early, at d23 of NGN1 differentiation, prompting the induced expression of *NGN1* which then drives the differentiation. The expression of NGN1 decreased after d23 and stayed at lower levels. It is worth noting that the peak of *NGN1* expression in small molecule differentiation is at d30, 10 days later than NGN1 differentiation which may indicate a slower developing speed for small molecule differentiation during these stages. In addition to that, the mRNA expression of *SOX10*, a classical neural crest cell marker, was downregulated in the iPSC-SNs compared to hiPSCs (Fig. 3E), whereas they exhibit a high expression of *TRKA* and *TRPV1* indicating the presence of nociceptor-like cells (Fig. 3F, G).

### Immunostaining confirms an increasing Na_V_1.7 expression in both differentiation methods

**Fig. 4:**
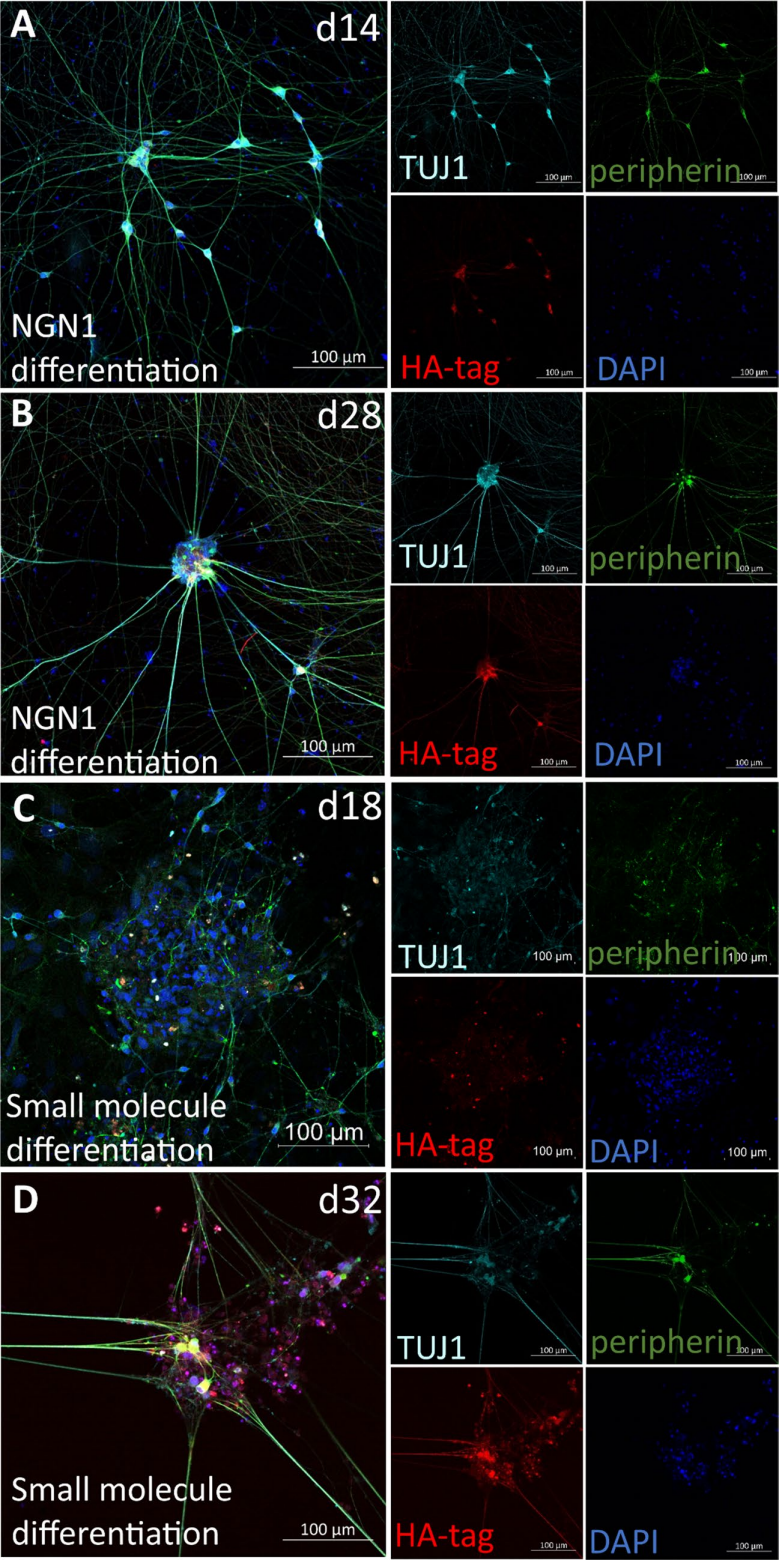
Early detection of Na_v_1.7 protein expression for two differentiation methods (confocal microscopy). **A**, **B** Immunostaining of iPSC-SNs of NGN1 differentiation: the presence of peripheral neuron marker TUJ1 (turquoise) and peripherin (green) and presence of HA-tag (red) at d14 (left) and d28 (right) with nuclei stained with DAPI (blue) are visible (scale bar 100 µm). **C**, **D** Immunostaining of iPSC-SNs of small molecule differentiation: the presence of peripheral neuron marker TUJ1 (turquoise) and peripherin (green) and presence of HA-tag (red) at d18 (left) and d32 (right) with the nucleus marker DAPI (blue) are shown (scale bar 100 µm). Three areas (either cluster of cells or more than five single cells per each area) of one coverslip for each differentiation were examined. And coverslips come from two time points of the same differentiation

Next, we asked whether the HA-tagged Na_V_1.7 protein expression is induced and detectable during differentiation of iPSC-SNs. We performed immunofluorescence staining to test for the HA-tag fusion peptide expression at d14 and d28 (NGN1 differentiation) and d18 and d32 (small molecule differentiation), respectively (Fig. 4A–D). Expression of HA-tagged Na_V_1.7 was detectable at all time points tested. The cellular localization changes over time: At the earliest time points stained, HA-tagged Na_V_1.7 is mainly localized in the soma, and at later time points, also neurites are stained positive, indicating an ongoing maturation affecting the subcellular localization of Na_V_1.7. HA-tagged Na_V_1.7-expressing cells co-express TUJ1, a neuron-specific class III beta-tubulin found in both the central as well as the peripheral nervous system and the expression of peripherin, a type III intermediate filament protein expressed in the neurons of the peripheral nervous system. For small molecule differentiation, expression of TUJ1 and peripherin is increasing in intensity at d32 compared to d18 (Fig. 4C, D), and the expression of TUJ1 and peripherin stays at high level at d14 and d28 of NGN1 differentiation (Fig. 4A, B), which shows ongoing maturation of peripheral sensory neurons during differentiation. Thus, we were able to generate a cellular system that reliably allows the staining of Na_V_1.7 during differentiation and maturation of sensory neurons.

### Na_V_1.7 cell surface localization occurs after 50 days of differentiation

In order to further track the temporal changes in the protein expression and localization of the HA-tagged Na_V_1.7, we monitored the surface expression of HA-tag at several time points during the time course of differentiation using immunofluorescence studies.

**Fig. 5:**
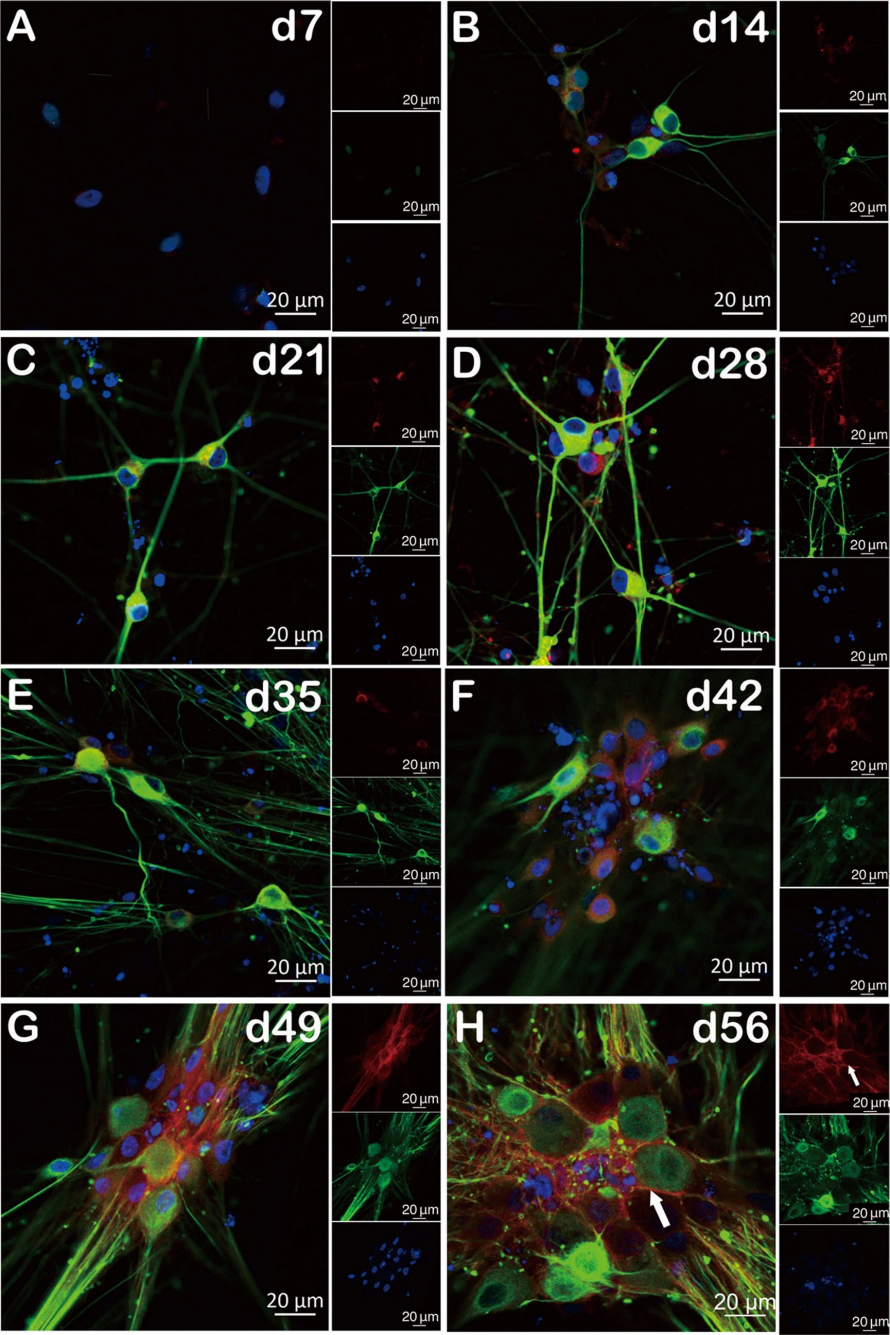
Detection of Na_V_1.7 protein surface expression at d56 during NGN-1 differentiation (confocal microscopy). **A**–**H** Double immunolabeled (peripherin (green) and HA-tag (red)) iPSC at d7 of differentiation (**A**) to mature sensory neuron ganglia–like structures at d56 of differentiation (**H**). The cell nuclei were stained with DAPI (blue). The distinct surface expression of HA-tag is marked with white arrows. All the data represents an overlay of peripherin (green) and HA-tag (red) staining (scale bar 20 µm). The representative pictures of d7–d28 were derived from two individual NGN1 differentiation and 6–10 single cells in total. Three cell clusters (containing more than five iPSC-SNs) from d35–d56 were examined from one NGN1 differentiation

We performed immunofluorescence staining to investigate the expression of HA-tagged Na_V_1.7 in NGN1 differentiation from a continuous long-term observation. We found the expression of peripherin is absent at d7 (Fig. 5A) but started from d14 (Fig. 5B) and enriched during differentiation (Fig. 5C–H). Similarly, there is no HA-tag expression at d7 of NGN1 differentiation (Fig. 5A); then, an increased expression of HA-tagged Na_V_1.7 starts from intracellular (Fig. 5B–G) to surface (Fig. 5H) during the time course of differentiation. It is worth noting that a clearly distinguishable surface expression of HA-tagged Na_V_1.7 appears at the borders of the peripheral neurons only at d56 (Fig. 5H) and was hardly detected at early time points. At this time point, the cells formed clusters, which impeded the detection of single cells. Thus, increase in signal intensity may also result from merging cells and not only from higher surface expression.

**Fig.6:**
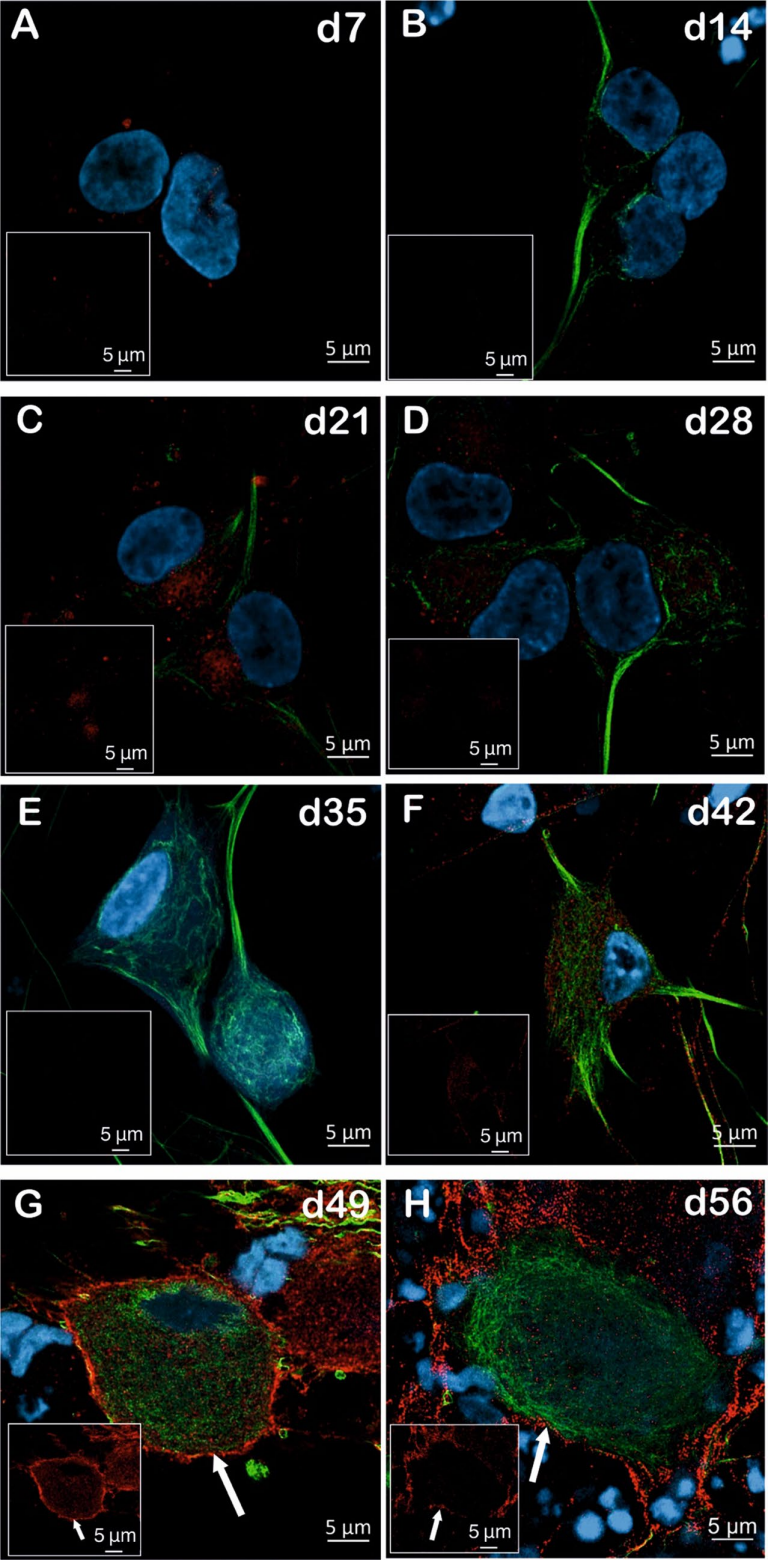
Na_V_1.7 protein surface expression is detectable at d49 during NGN-1 differentiation (Airyscan microscopy). **A**–**H** Images of double immunolabeled (peripherin (green) and HA-tag (red)) iPSC-SNs from d7 of differentiation (**A**) to mature sensory neurons at d56 of differentiation (**H**) acquired using a LSM 980 with Airyscan 2. The cell nuclei were stained with DAPI (blue). The distinct surface expression of HA-tag is marked with white arrows. All the data represents an overlay of peripherin (green) and HA-tag (red) staining. Insets show representative picture of labeled HA-tagged Na_V_1.7 from each time point and a clear surface localization of Na_V_1.7 at d49 of differentiation. White arrow shows the surface expression of HA-tagged Na_V_1.7 (scale bar 5 µm). The representative pictures are taken from two individual NGN1 differentiations, and 6–10 single cells from two coverslips per time point have been examined in total

Given that the surface expression of HA-tagged Na_V_1.7 at d56 of NGN1 differentiation is hard to determine unequivocally, we applied higher-resolution confocal microscopy to distinguish the membrane expression of HA-tagged Na_V_1.7 on iPSC-SNs. Therefore, representative confocal images of double immunolabeled (peripherin and HA-tag) HA-tagged Na_V_1.7 iPSC-SNs were taken with a LSM 980 with Airyscan 2 microscope. As Fig. 6A, B shows, there is no expression of HA-tagged Na_V_1.7 at d7 and d14, and the intracellular expression becomes visible from d21 on and remains unchanged until d42 (Fig. 6C–F). Importantly, a distinguishable surface expression of HA-tagged Na_V_1.7 at the borders of the peripheral neurons is clear at d49 and d56 (Fig. 6G, H). Z-stacking videos of d49 and d56 show reliable detection of surface localization of HA-tagged Na_V_1.7 at different focal distances (Supplementary Video V1-2). A distinguishable surface expression of HA-tagged Na_V_1.7 exists in 55.5% of iPSC-SNs at d49 and 50% at d56 (Table 1, Fig.S3-4).

**Table 1:** Summary of the expression level of HA-tagged Na_V_1.7 in NGN1 differentiation, as determined by microscopy

Time point	Peripherin expression	HA-tag expression	Distinct surface expression of HA-tag	Proportion of surface expression in neurons per neurons investigated
d7	**×**	**×**	**×**	0/6
d14	**√√√**	**√**	**×**	0/10
d21	**√√√**	**√**	**×**	0/8
d28	**√√√**	**√**	**×**	0/8
d35	**√√√**	**√√**	**×**	0/7
d42	**√√√**	**√√**	**×**	0/8
d49	**√√√**	**√√√**	**√√√**	5/9
d56	**√√√**	**√√√**	**√√√**	4/8

**×** indicates no expression; **√** indicates low expression; **√√** indicates medium expression; **√√√** indicates high expression

**Fig. 7:**
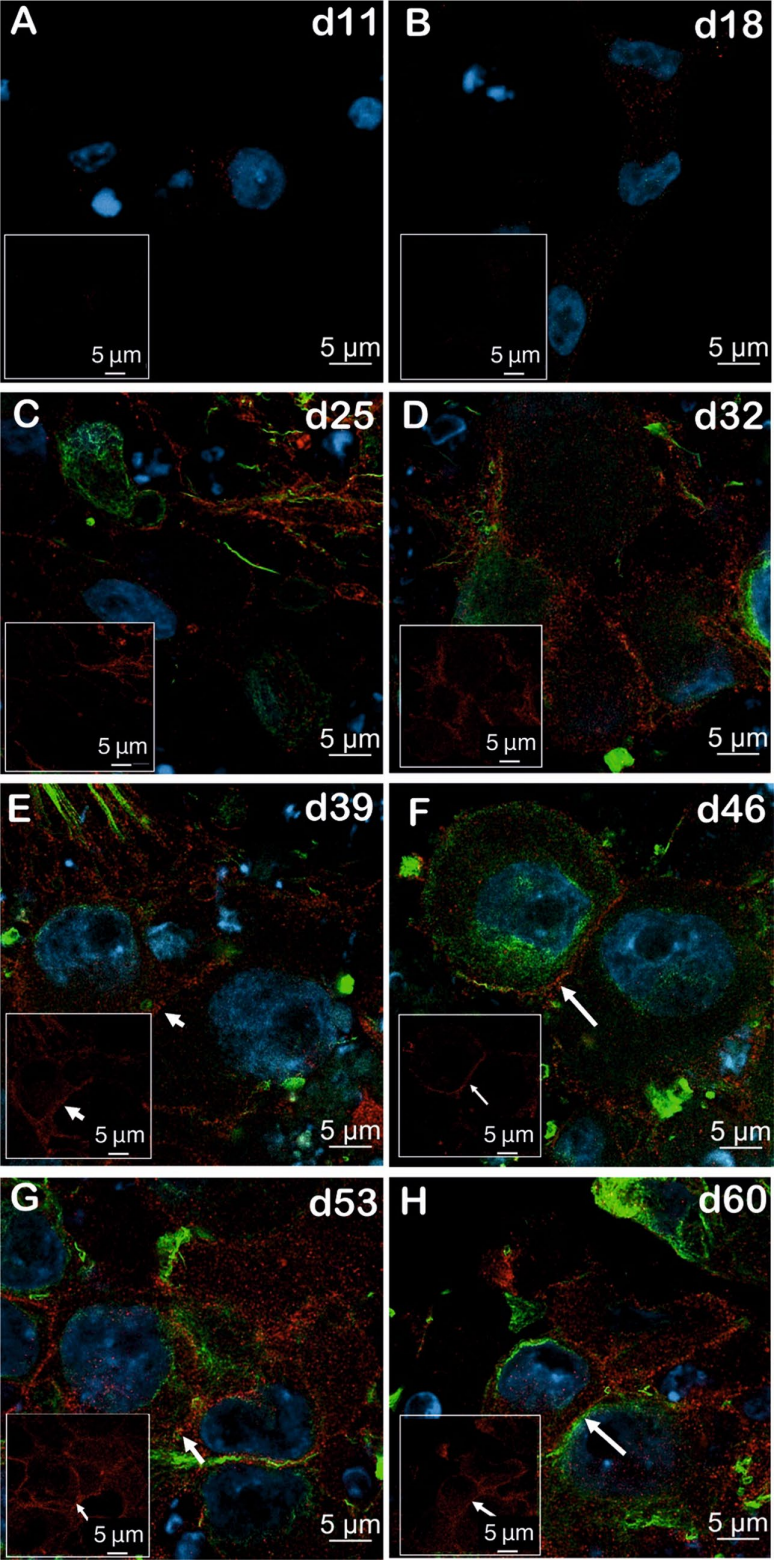
Na_V_1.7 protein surface expression during small molecule differentiation starts at d39 and is clearly visible from d53 onwards (Airyscan microscopy). **A**–**H** Images of double immunolabeled (peripherin (green) and HA-tag (red)) iPSC-SNs from d11 of differentiation (**A**) to mature sensory neurons at d60 of differentiation (**H**) acquired using a LSM 980 with Airyscan 2. The cell nuclei were stained with DAPI (blue). The detectable expression of the HA-tag is marked with white arrows. Data represents an overlay of peripherin (green) and HA-tag (red) staining. The images in the white box showed representative pictures of expression of HA-tagged Na_V_1.7 from each time point and a clear surface localization of Na_V_1.7 from d53 to d60 of differentiation. White arrow shows the surface expression of HA-tagged Na_V_1.7 (scale bar 5 µm). The representative pictures are taken from two individual coverslips iPS-SNs derived from one small molecule differentiation, and four to seven areas per time point have been examined in total. Another small molecule differentiation was repeated, and pictures (supplementary fig. S5) were taken with the same procedures as in Fig. 6. Three to six areas have been examined

We also performed immunofluorescence stainings from d0 (hiPSCs) to d60 of nociceptors differentiation during small molecule differentiation and imaged either with normal confocal or Airyscan microscope. There is no HA-tagged Na_V_1.7 expression in hiPSCs (Fig.S2A); then, Na_V_1.7 enriched intracellularly from d11 to d32 through time course of differentiation (Fig. 7A–D, Fig.S2B-E). The surface localization of HA-tag starts to be detectable from d39 and becomes clearer at d46 of differentiation (Fig. 7E, F, Fig.S2F-G). After that, a visible and distinguishable surface expression of the HA-tag was clearly evident at d53 and d60 of differentiation (Fig. 7G, H; Fig.SHF-I), which corroborates with the data shown in McDermott et al. But due to iPSC-SNs derived from small molecule differentiation form ganglion-like clusters, it is really hard to calculate the percentage of those cells which show definite surface localization of Na_V_1.7 even from Airyscan images (Fig. 7, Fig. S5). At the same time, compared to d0, d11, and d18, the expression of peripherin from d25 to d60 is increasing through the time course of differentiation which indicates the maturation of nociceptors (Fig. 7C–H, Fig.S5C-H).

## Discussion

In this study, we show that electric activity of iPSC-derived sensory neurons occurs long before surface expression of Na_V_1.7. We compare the surface expression of Na_V_1.7 during iPSC differentiation into sensory neurons using two previously published protocols: small molecule or forced NGN-1 expression. We used an iPSC line in which Na_V_1.7 is labeled by an HA-tag and monitored the time course of Na_V_1.7 expression and sub-cellular localization using Airyscan microscopy. We found that at least 50 days are necessary for reliable Na_V_1.7 surface expression for both differentiation approaches. Since pluripotent stem cells have been established as readily available resources, it became possible to generate somatic human cell types in the lab, which are not easily available from other sources. Protocols to generate peripheral sensory neurons from hiPSCs have paved the way for new, more human-relevant test systems. The nociceptor differentiation of the HA-tagged Na_V_1.7 hiPSCs was successfully performed with two well-established protocols based on fundamentally different approaches: one is based on small molecule–driven differentiation, and the other is initiated by viral forced overexpression of NGN1. Small molecule approaches for sensory neuron differentiation yield a mixed population of neurons with characteristics similar to those observed in primary DRGs, including gene expression, responses to noxious stimuli, and functional expression of ion channels and receptors [5, 37]. However, the protocol has also been shown to lead to variations in the resulting neuronal population, including the presence of proliferative non-neuronal cells [31]. Thus, we included a recently published protocol into our study, relying on forced expression of NGN1 to synchronize culture and drive neuronal maturity [30]. Immunofluorescence and qRT-PCR studies confirmed the expression of the pan-neuronal markers TUJ1 and the peripheral neuronal marker peripherin at different time points of both differentiation protocols.

In vivo, NGN1 expression regulates the development of NTRK1 (coding for TRKA-expressing) nociceptors [1, 6]. Our data compares to previous studies [30] showing that overexpression of NGN1 is sufficient to drive nociceptor development from NCLCs [30]. In our hands, *TRKA* gene was significantly upregulated compared to the hiPSCs. We also detected an increased expression of *TRPV1* by qRT-PCR confirming the maturation of nociceptors during differentiation [24, 30]. A key marker in identification of nociceptive sensory neurons is *SCN9A*, which codes for the Na_V_1.7 sodium channel and is highly expressed in DRGs. In our model, the expression of *SCN9A* was significantly upregulated during maturation of nociceptors derived from both protocols. From some studies, expression of these nociceptor markers in hiPSC-derived nociceptors, like *SCN9A* and *SCN10A*, was lower compared to primary DRGs [27]. An extended analysis with recently published datasets could provide further insights with regard to differences between maturity of hiPSC-derived nociceptors and human DRGs. Recently, human single-nucleus RNA-seq datasets have been published [25], and a spatial transcriptomics analysis of human DRGs has been described [35], which will help to compare datasets and understand the usefulness of the here presented model system in more depth. The exploratory data analysis tool NOCICEPTRA provides a platform to characterize iPSC-SNs differentiation stages, ion channel expression, and to analyze miRNAs of interest [14, 38]. It is interesting to evaluate the maturity of nociceptors derived in vitro from hiPSCs with primary DRGs on a single-cell level.

Our data detect gene expression of *SOX10* for hiPSCs, and the signal declines during both differentiation methods, albeit more strongly in the small molecule approach. *SOX10* is normally seen in immature neurons and NCLCs, which are produced with our differentiation strategies, where we start with a mix of neurons and NCLCs, and in time, *SOX10* expression declines with the increase of maturity of the generated sensory neurons. The more enhanced decline of *SOX10* expression in the small molecule approach may either indicate a more mature population of sensory neurons or may also reflect the appearance of non-neuronal cell types, which we observed, and which was described in the literature before.

Until a few years ago, the major models to study peripheral pain-related neuropathies were experimental animals. The in vitro studies mainly focused on structural defects or molecular dysfunctions, and the main test systems for this were rodent neurons or heterologous expression systems. Robust quantitative studies on human nociceptor modulation are, thus, quite limited, and iPSC-SNs offer a versatile tool to study nociceptor physiology and pathophysiology. In vivo studies often assess behavioral outcome measures that are the result of a complex integration of peripheral, central, and glial cell–type activities [26]. In such cases, specific mechanisms or receptors are hard to assess.

Like most integral membrane proteins, the membrane expression of functional sodium channels is crucially controlled by both cell-type specific and common components of the protein generation and trafficking machinery. According to their tissue function, excitable cells display various morphologies associated with specialized electrical membrane domains. Studying the transport of Na_V_ channels to the cell surface and how neurons accomplish and maintain such a polarized distribution is still under investigation. In our study, we observed that the RNA expression of Na_V_1.7 was at a high level around 30 days of differentiations in both protocols, but Na_V_1.7 protein was still distributed equally intracellularly. This conforms to a procedure of translation in rough endoplasmic reticulum, biosynthesis in both endoplasmic reticulum and Golgi apparatus, and a transport via the vesicular system. Translation causes an increase in RNA level; then, the protein synthesis follows. In both differentiation approaches, *HA-tag* mRNA was half of *SCN9A* mRNA, reflecting the heterozygous genotype of our iPSC line. HA-tag location along the neurites preceded the cell surface expression in our data. The specific underlying mechanisms for the delivery of Na_V_1.7 from intracellular to surface still need to be clarified.

Channel kinetics position Na_V_1.7 as a threshold channel amplifying sub-threshold depolarizations in nociceptor terminals and by virtue conferring a critical role for Na_V_1.7 in action potential electrogenesis [18, 19]. Thus, knowing the time point of its expression on the membrane of iPSC-SN will be necessary to guide follow-up studies investigating its pharmacology and pathophysiological role. Indeed, iPSC-based in vitro test methods have been repeatedly used to measure the effects of Na_V_1.7 on nociceptors’ function [18, 19, 23]. However, there is still a need for studies that employ iPSC-derived nociceptor cultures to assess alterations of membrane expression of Na_V_1.7 protein endpoints. The cell system, together with the endpoints we characterized, may help to fill this gap for physiological and electrophysiological studies.

From our study, the surface expression at the borders of the neuronal cells become gradually distinguishable from d49 until d56 in NGN1-derived differentiation, and a clear maximum distinguishable surface expression of Na_V_1.7 was observed at d49 with this protocol. D49 could be recognized as a standard optimal time point for functional characterization of Na_V_1.7 in NGN1-driven differentiated nociceptors owing to the presence of Na_V_1.7 on the surface of the nociceptors. Similarly, in small molecule–driven differentiation, the surface expression becomes gradually detectable from d39 until d60, with a surface localization at d60 in our differentiations. Despite different differentiation strategies at early phases and potentially also differences in the speed of differentiation, cells always need around 50 days for Na_V_1.7 to be expressed at the surface. We did not investigate time points after day 60, and there might be a better surface localization which needs to be clarified in future studies.

Our electrophysiology data show that small molecule differentiation of iPSC-SNs leads to neurons that start to become electrically active at day 22 and the spontaneous action potential firing continuously increases until day 40 of differentiation in three independent differentiations. Thus, at day 22 the latest, enough functional sodium channels are present for the generation of measurable action potentials. These sodium channels are likely not comprised of Na_V_1.7, but of other neuronal subtypes, such as Na_V_1.1, Na_V_1.2, or Na_V_1.6 [38]. Thus, studies focusing on the function or pharmacological effects of a block of Na_V_1.7 need to be performed following day 49 of differentiation.

In our system, the HA-tag is introduced in only one allele of SCN9A, while the other allele remains unchanged. Consequently, the function of Na_V_1.7 is likely to be unchanged, and thus the system lends itself for future functional studies. It may still be possible that the tagged allele’s function is impaired. As the parents of CIP patients often carry a heterozygous loss-of-function mutation in one of their alleles and are nevertheless clinically without phenotype, it is highly likely that our system represents a functional physiological state, even if the very small HA-tag may affect Na_V_1.7 function. We here show that HA-tagging of Na_V_1.7 allows in detail study of sodium channel trafficking and detection of surface expression. This approach can now also be applied to other ion channels of interest, especially those for which good antibodies are sparse and which are closely linked to disease, such as Na_V_1.8 and Na_V_1.9.

To summarize, we have undertaken a detailed staining analysis of the expression and localization of Na_V_1.7 in hiPSC-derived nociceptors differentiated by two distinct protocols. We showed that the Na_V_1.7 HA-tagged hiPSC-derived sensory neurons expressed hallmark neuronal and nociceptive markers, confirming their sensory neuron identity. The determination of the time points of surface expression of Na_V_1.7 showed maximum distinguishable presence in the membrane of HA-tagged Na_V_1.7 at d49 of NGN1-derived neurons, which according to our data can be used as an optimal time point for Na_V_1.7 studies in the future. In addition to this, our data show that it takes at least 50 days of differentiation to achieve robust surface expression of Na_V_1.7 for both differentiation strategies investigated here. The correlation of the above determined time points of distinguishable surface expression of the HA-tagged Na_V_1.7 is important to completely validate it as an in vitro model for functional characterization of Na_V_1.7, e.g., for drug screening.

## Methods

### Generation and maintenance of hiPS cells

hiPSCs (B1 clone 102.2) used for HA-tagging were kindly provided by Prof. Dr. Wolfgang Wagner (Institute for Stem Cell Biology, Uniklinik RWTH Aachen). The iPSCs were reprogrammed from mesenchymal stromal cells from a healthy female donor by electroporation of the plasmids pCXLE-hSK, pCXLE-hUL, and pCXLEhOCT3/4-shp53.

hiPSCs were cultured in StemMACS™ iPS-Brew XF (Miltenyi Biotec) on Matrigel- (Corning) or Geltrex (Life Technologies)-coated dishes under humidified atmospheric conditions (37 °C, 5% CO_2_) following the manufacturer’s recommendations. Regular passaging was performed 1–2 times per week in a ratio of 1:5 to 1:10 using 0.5 mM EDTA in PBS (Life Technologies). Medium was changed daily. Testing for mycoplasma contamination was performed regularly using the mycoplasma detection kit “MycoSPY” (Biontex) according to instructions.

### Generation of HA-tagged Na_V_1.7 hiPSC line

The DNA sequence coding for the HA-tag was inserted at the C-terminus of the SCN9A gene by CRISPR/Cas9. Briefly, a ribonucleoprotein (RNP) complex of HiFi Cas9 nuclease (IDT) and gRNA (crRNA + tracrRNA, both from IDT) targeting the C-terminus of the SCN9A gene was delivered to a single-cell suspension of iPSCs with the Neon™ Transfection System 100 µl Kit (Invitrogen, 1 pulse, 30 ms, 1300 mV). A 144-nucleotide single-strand DNA oligo (IDT) was used as a homology-driven repair (HDR) donor template. The HDR donor template consisted of 5′ and 3′ homology arms flanking the CRISPR/Cas9 target site and the HA-tag coding sequence followed by a novel STOP codon. Prior to transfection, cells were treated with 5 µM HDR enhancer (IDT) and 10 µM ROCK Inhibitor (Y-27632, StemCell Technologies) for 1 h. iPSC single-cell suspension was generated by Accutase (PAN-Biotech) treatment for 10 min at 37 °C. After transfection, cells were seeded on Matrigel (Corning)-coated 6-well plates (Greiner) in iPS-Brew (Miltenyi Biotec) supplemented with 1 × CloneR™ (StemCell Technologies). For the isolation of clonal iPSC lines, a single-cell suspension of the transfected cells was seeded at low density on Matrigel-coated 10-cm dishes in StemMACS iPS-Brew initially supplemented with 10 µM Y-27632 (StemCell Technologies). iPSC colonies derived from a single cell were manually picked and expanded for further analysis. Screening for HA-tag correct integration was performed by PCR and Sanger sequencing. Oligonucleotide sequences are provided in Supplementary Table 3.

For that, genomic DNA of clonally expanded hiPSCs was isolated using the NucleoSpin DNA Kit (Macherey–Nagel, Germany) according to the manufacturer’s instructions. The HA-tagged Na_V_1.7 target region was PCR-amplified with forward, reverse and the flanking primer mentioned in Supplementary Table 1 (Eurofins Genomics). The PCR (OneTaq® Quick-Load® DNA Polymerase, all materials obtained from New England Biolabs) was carried out under the following conditions: A 5-min preincubation period at 94 °C followed by a three-step amplification: denaturation of the amplicon was done at 94 °C for 30 s, primer annealing at 50 °C for 45 s, and finally elongation at 68 °C for 30 s. This cycle was repeated 35 times. The PCR products were separated with a 1.5% agarose gel (Invitrogen, USA) supplemented with RedSafe Nucleic Acid Staining Solution (iNtRON Biotechnology, South Korea) run in TAE buffer (Roth, Germany) at a constant voltage of 90 V over a period of 30 min. The images were taken in the gel documentation system Quantum (Vilber). The HA-tag-specific PCR is illustrated in schematic Figure S1. The PCR amplified a product specific for the HA-tag (300 bp) and an internal control (440–473 bp). Successful integration of the HA-tag was confirmed by the presence of both products.

To further confirm the presence of the HA-tag, the PCR product amplified with forward and flanking primers were purified with peqGOLD MicroSpin Cycle Pure Kit (VWR Peqlab, USA) according to the manufacturer’s instructions and sent for sequencing (Eurofins Genomics, Germany).

### Flow cytometry

For flow cytometry, cells were dissociated with Accutase (Sigma-Aldrich) for 5 min to create a single-cell suspension, and 1 × 10^5^ cells were stained with Anti-Human TRA-1–60 and Anti-human SSEA-4 antibodies (Supplementary Table 2). Staining was carried out for 30 min at 4 °C. Cells were then washed twice with 10% FBS in PBS and centrifuged at 500 × g for 5 min. Measurements were performed with the BD FACS Canto II (Becton Dickinson Biosciences, Germany), and FCS Express software (FCS Express, USA) was used for data analysis.

### Small molecule–driven differentiation of sensory neurons

Differentiation of hiPSCs was carried out as described previously (Neureiter et al., 2022). The protocol consists of a nociceptor induction phase of 10 days and a maturation phase of 5–6 weeks (Fig. 1A).

To start the differentiation, 2 × 10^5^ cells/cm^2^ were seeded to Geltrex (Life Technologies, USA)-coated dishes in StemMACS iPS-Brew (Miltenyi Biotec) in the presence of 10 µM Y-27632 (StemCell Technologies). After 24 h, cells reached 90–100% confluence, and the nociceptor induction phase was started (= d0) by changing the medium to KSR medium (DMEM/F12, 15% knockout serum replacement, 200 µM l-glutamine, 1 × MEM-NEAA, 100 µM β-mercaptoethanol (all from Life Technologies, USA), 100 U/ml penicillin, and 100 µg/ml streptomycin (Sigma-Aldrich, USA)) supplemented with 10 µM SB431542 (Sigma-Aldrich, USA) and 1 µM LDN-193189 (Miltenyi Biotech, Germany). Both small molecules were used until d6. From d2 on, 10 µM DAPT (Tocris), 10 µM SU5402 (Sigma-Aldrich), and 3 µM Chir99021 (Tocris) were added to the medium. From d4 to d10, the KSR medium was gradually transitioned to N2/B27 medium containing 50% DMEM/F12, 50% Neurobasal, 1 × N2 supplement, 1 × B27 supplement, 200 µM l-glutamine, 1 × MEM-NEAA (all from Life Technologies, USA) and 100 U/ml penicillin and 100 µg/ml streptomycin (Sigma-Aldrich, USA) in 25% increments.

On d10, for nociceptor maturation, cells were dissociated with Accutase (Sigma-Aldrich) and reseeded to glass coverslips coated with 15 µg/ml poly-l-ornithine (Sigma-Aldrich, USA), 15 µg/ml laminin (Sigma-Aldrich, USA), and 15 µg/ml fibronectin (Life Technologies, USA) in a density of 50,000 cells/cm^2^ in Neurobasal medium (Neurobasal, 1 × B27 supplement, 200 µM l-glutamine (all from Life Technologies), 100 U/ml penicillin, 100 µg/ml streptomycin (Sigma-Aldrich)). The Neurobasal medium contained 20 ng/ml human NGF-beta (Tocris, Bristol, UK), human BDNF, human GDNF (PeproTech, Germany), 200 µM ascorbic acid (Sigma-Aldrich, USA), and 0.5 mM dbcAMP (StemCell Technologies) for the entire maturation phase of nociceptors. Until d17, 10 µM DAPT and 10 µM SU5402 (both from Tocris) were added as well. The medium was changed every 3–4 days. 500 ng/ml laminin (Sigma-Aldrich, USA) was included in long-term maintenance medium from day 25 onward.

### NGN1-driven differentiation of sensory neurons

The differentiation of iPSC-SNs by forced expression of NGN1 was described previously and contains two steps: first, the generation of neural crest–like cells (NCLCs) and second, the differentiation of sensory neurons (Schrenk-Siemens et al. 2022).

For the differentiation of NCLCs, iPSCs were detached with 0.5 mM EDTA (Life Technologies, USA) as large clusters and transferred to an uncoated 10-cm dish (Greiner) with sphere medium (50% DMEM-F12 medium, 50% Neurobasal, 0.5 × N2, 0.5 × B27 supplement, 200 µM l-glutamine (all from Life Technologies, USA), 2.5 µg/ml insulin (CSBio, USA), 10 ng/µl EGF, and FGF (PeproTech, Germany)). 10 µM Y-27632 (StemCell Technologies) was added for the first 24 h of sphere culture. The medium was changed the next day, followed by every 2nd day medium change. Spheres started to attach spontaneously between day 5 and day 10 and NCLCs migrated out. On that day, all spheres were aspirated and NCLCs collected with Accutase (Life Technologies). Collected NCLCs were frozen in 90% sphere medium and 10% DMSO or directly plated for the second step of differentiation.

To generate iPSCs-SNs, 50,000 NCLCs/cm^2^ were seeded to polyornithine-, laminin-, and fibronectin (all used at a concentration of 15 µg/ml)-coated dishes in sphere medium without EGF and FGF. After 16–24 h, the medium was supplemented with 10 mM HEPES (Thermo Fisher Scientific, USA) and 8 µg/ml protamine sulfate (Sigma-Aldrich), and two lentiviruses with a MOI = 0.5 were added. One lentivirus carries the coding sequence for constitutive expression of rtTA and the other for the expression of NGN1, GFP, and a puromycin resistance under the control of a tetracycline response element. The next day (= d0), cells were washed three times with PBS and cultured in sphere medium containing 10 ng/ml BDNF, GDNF, and NGF (all from PeproTech, Germany). 2 µg/ml doxycycline (Sigma-Aldrich, USA) was added until day 10 after virus infection to induce the expression of NGN1, GFP, and the puromycin resistance. 10 µg/ml puromycin (Invitrogen, USA) was added at day 3 for 24 h to select for transduced cells only. At d5, cells were passaged to glass coverslips coated with 15 µg/ml poly-l-ornithine (Sigma-Aldrich, USA), 15 µg/ml laminin (Sigma-Aldrich, USA), and 15 µg/ml fibronectin (Life Technologies, USA) in a density of 30,000 cells/cm^2^. Starting from d10, the medium was changed every 2–3 days but without doxycycline until analysis.

### Lentivirus generation for forced expression of NGN1

Lentiviral particles were produced in HEK293T cells cultured in DMEM (Life Technologies) and 10% FBS (Sigma-Aldrich) (defined as 293 T medium). For lentivirus production, the three helper plasmids pMD2.G (encoding for VSV-G), pRSV-Rev (encoding for Rev), and pMDLG/pRRE (encoding for the lentiviral GAG-Pol) and the plasmid FUW-Tet-ON-Ngn1-P2A-EGFP-T2A-PuroR or FUW-rtTA were co-transfected into 293 T cells by calcium phosphate. Next morning, the medium was exchanged. 48h and 72 h after co-transfection, the lentiviral particles containing supernatant was harvested and precipitated with 80 µg Polybrene and 80 µg chondroitin sulfate (both obtained from Sigma-Aldrich) at 37 °C for 20 min followed by centrifugation at 3800 g for 20 min. The virus was resuspended in DMEM containing 20 mM HEPES and stored at − 80 °C until use.

To determine the lentiviral titre, 1 × 10^5^ 293 T cells were seeded and 6–8 h later transduced with a defined volume of both lentiviruses. Transduction was carried out in 293 T medium, 8 µg/ml protamine sulfate and 10 mM HEPES. The medium was exchanged the next day, and 2 µg/ml doxycycline was added to the 293 T medium. The percentage of transduced cells was determined based on the GFP signal and measured by flow cytometry (BD FACS Canto II). Analysis was performed with FCS Express 7, and the number of infectious units/ml was calculated.

### RNA isolation and cDNA synthesis

Cells were detached by Accutase and centrifuged for 5 min at 200 g. RNA of the obtained cell pellets were further isolated with the NucleoSpin RNA Kit (Macherey–Nagel, Germany) according to the manufacturer’s instructions and eluted with 40 µl of RNAse-free water. The RNA concentration and purity were determined with the NanoDrop 2000c (Thermo Fisher Scientific, USA). For the following cDNA synthesis, 1 µg RNA was used for reverse transcription. The cDNA synthesis followed the SensiFAST cDNA Synthesis Kit (Bioline, USA) as described by the manufacturer and was performed in a peqSTAR Thermocycler (VWR Peqlab, Germany). Extracted cDNA was stored at − 20 °C or directly used for qRT-PCR.

### Quantitative real-time PCR

The SensiMix SYBR No-ROX Kit (Bioline, USA) was used according to the manufacturer’s instructions, and quantitative real-time PCR was performed on a Rotor-Gene Q real-time cycler (Qiagen, Germany) or on CFX Opus 96 Real-Time PCR Systems (Bio-Rad, USA). A 10-min preincubation period at 95 °C followed by an amplification cycle: at 95 °C for 15 s, at 60 °C for 1 min, and repeated 40 times. Primers (Eurofins Genomics, Germany) in this study are listed in Supplementary Table 1. Obtained data were calculated based on the 2^–∆∆C^^t^ method where data were normalized to the Ct-value of the housekeeping gene GAPDH of the same sample and depicted relative to the respective gene expression of hiPSCs.

### Immunofluorescence staining and microscopy

Cells were grown on polyornithine/fibronectin/laminin-coated glass coverslips and fixed with cold 4% paraformaldehyde (PFA) (Roth, Germany) for 10 min at room temperature (RT). PFA was removed, and cells were washed three times with PBS for 5 min, permeabilized with 0.1% Triton X-100 (Sigma-Aldrich, USA) for 15 min, and blocked with blocking solution (5% goat serum in PBS) for 1 h at RT. The blocking solution was aspirated, and 200 µl of primary antibodies diluted in blocking solution was added and incubated at 4 °C overnight. Next day, samples were washed three times with PBS for 5 min, and the secondary antibodies diluted in blocking solution were added for 1 h at RT protected from light. Samples were washed again three times with PBS. 2 drops/ml of NucBlue Fixed Cell ReadyProbes Reagenz (DAPI; Life Technologies) were added during the second wash step. Finally, coverslips were mounted on glass slides with Prolong™ glass antifade mountant and stored at 4 °C protected from light.

Fluorescence images were acquired with an inverted LSM 710 confocal microscope (Zeiss, Germany) using ZEN black software (Zeiss, version 2012 SP1) and a Plan-Apochromat 63 × /1.4 NA oil objective (Zeiss, Germany) or EC Plan-Neofluar 40 × /1.3 oil objective (Zeiss, Germany). High-resolution images were acquired with an inverted LSM 980 with Airyscan 2 (Zeiss, Germany) using ZEN blue software (Zeiss, version 3.6) and a Plan-Apochromat 40 × /1.3 NA oil objective (Zeiss, Germany). For detection of peripherin and HA-tag in high resolution, the Airyscan SR mode was used. The images were processed with Airyscan Joint Deconvolution. DAPI was detected with a confocal PMT detector and processed using the LSM Plus software module.

All primary and secondary antibodies used are listed under Supplementary Table 2.

## Multi-electrode array

For multi-electrode array recordings (MEA recordings), 100,000 cells were seeded on polyornithine-, laminin-, and fibronectin-coated MaxTwo 6-Well MEA plates (MaxWell Biosystems) at d10 of differentiation and further differentiated according to the small molecule–driven differentiation protocol. Cells were recorded every 2–3 days starting at d13 using the MaxTwo system and MaxLab Live software (MaxWell Biosystems). The recording was performed with the following settings: Gain 512 × , HPF ~ 300 Hz, and spike threshold 5.0. The Activity Scan was carried out with the checker board mode, recording 13,300 electrodes in 13 configurations and 45 s/configuration. The analysis was performed with MaxLab Live analysis tool. A firing rate threshold of 0.1 Hz, amplitude threshold of 20 µV, and an interspike interval threshold of 100 ms were applied to detect active electrodes. The data were exported, and the mean firing rate per well and the standard deviation were extracted and plotted in OriginPro 2019b.

### Statistical information

The samples for measurement of qRT-PCR were all independent replicate (independent differentiation) calculated by the mean of independent experiments ± standard error of mean (SEM). Statistical differences between iPSC-SN of different time points with hiPSCs were evaluated using two-way ANOVA analysis via Graph Prism 7.

### Supplementary Information

Below is the link to the electronic supplementary material.

Supplementary file1 (AVI 15263 KB)

Supplementary file2 (AVI 12488 KB)

Supplementary file3 (DOCX 11122 KB)

## Data Availability

All data are available upon request.

## Resource availability

Further information and requests for resources and reagents should be directed to and will be fulfilled by the corresponding author, Angelika Lampert (alampert@ukaachen.de).

## Data and code availability

No standardized datatypes and code were generated. All data are available from the corresponding authors under request.

## Materials availability

This study did not generate new unique reagents.
