# Supplementary material for: The pain target NaV1.7 is expressed late during human iPS cell differentiation into sensory neurons as determined in high-resolution imaging
Source: Pflugers Arch. 2024 Mar 27;476(6):975–92. doi: 10.1007/s00424-024-02945-w (PMC11139713; doi:10.1007/s00424-024-02945-w)
Supplement: Supplementary file 3 — Supplementary file3 (DOCX 11122 KB) [file 424_2024_2945_MOESM3_ESM.docx]

# Supplementary Figures


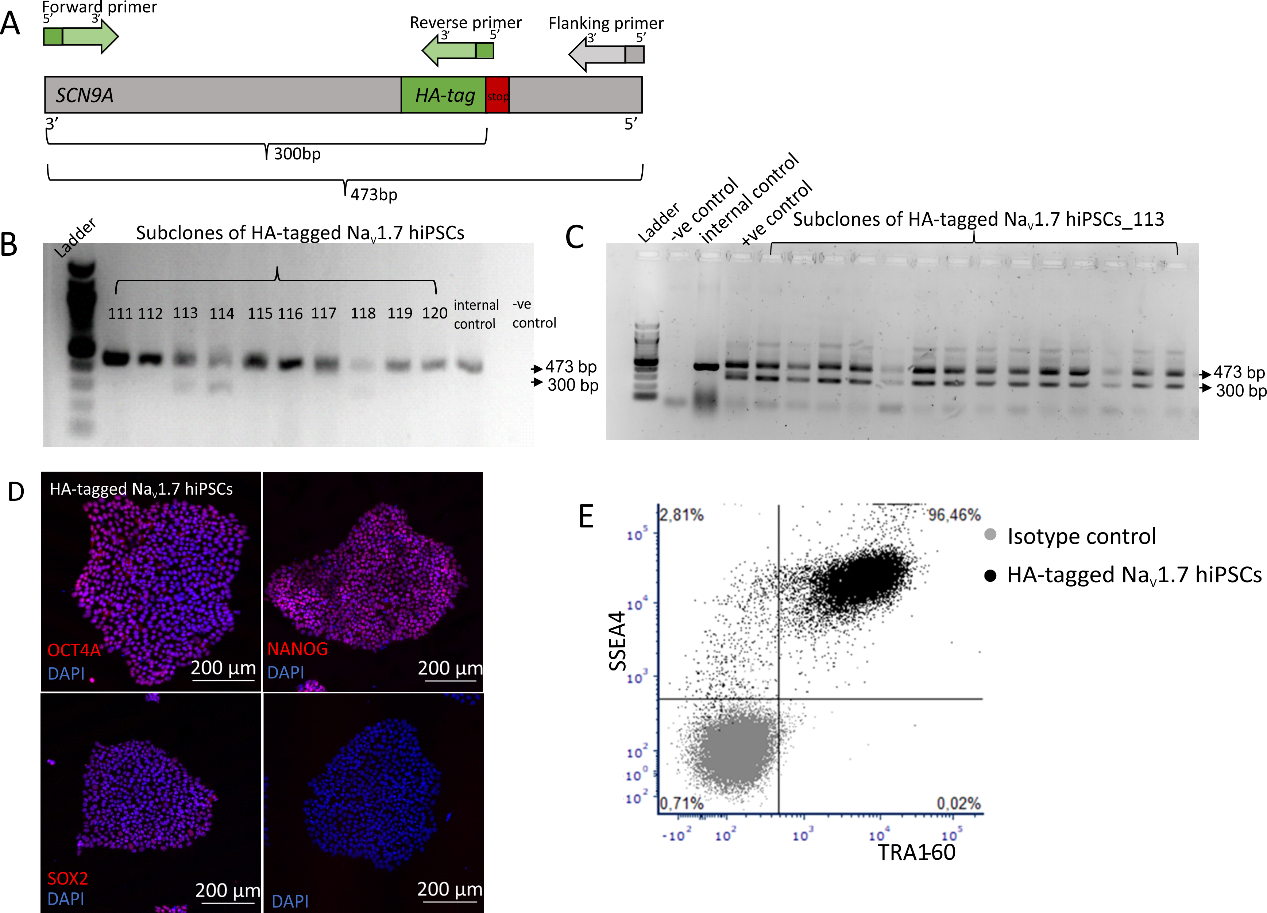


**Fig.S1** Detectable HA-tag presents on both gel electrophoresis of subclones and chosen subclones express pluripotency markers in immunostaining and FACS. **A**. Graphical representation of the HA-tag specific PCR. Forward, Reverse and Flanking primers were designed accordingly for the HA-tag specific PCR. The PCR product contains two DNA fragments: the longer one (473bp) is the end of the coding sequence of *SCN9A* gene whereas the shorter one(300bp) is specific to the HA-tag. **B-C**.Agarose gel electrophoresis of HA-tag specific PCR products shows two distinct bands at 300bp and 473bp. -ve control: negative control; +ve control: positive control. **D-E**. HA-tagged Na_V_1.7 hiPSCs maintain their pluripotent state after the CRISPR mediated genome editing and subcloning process. **D** showed the expression of pluripotency markers (OCT4A, SOX2, NANOG) (red) in HA-tagged Na_V_1.7 hiPSCs. The cell nuclei are stained in blue with DAPI. (scale bar: 200µm) **E** showed the expression of surface markers (TRA-1-60 and SSEA-4) in 96.89% of live cells.


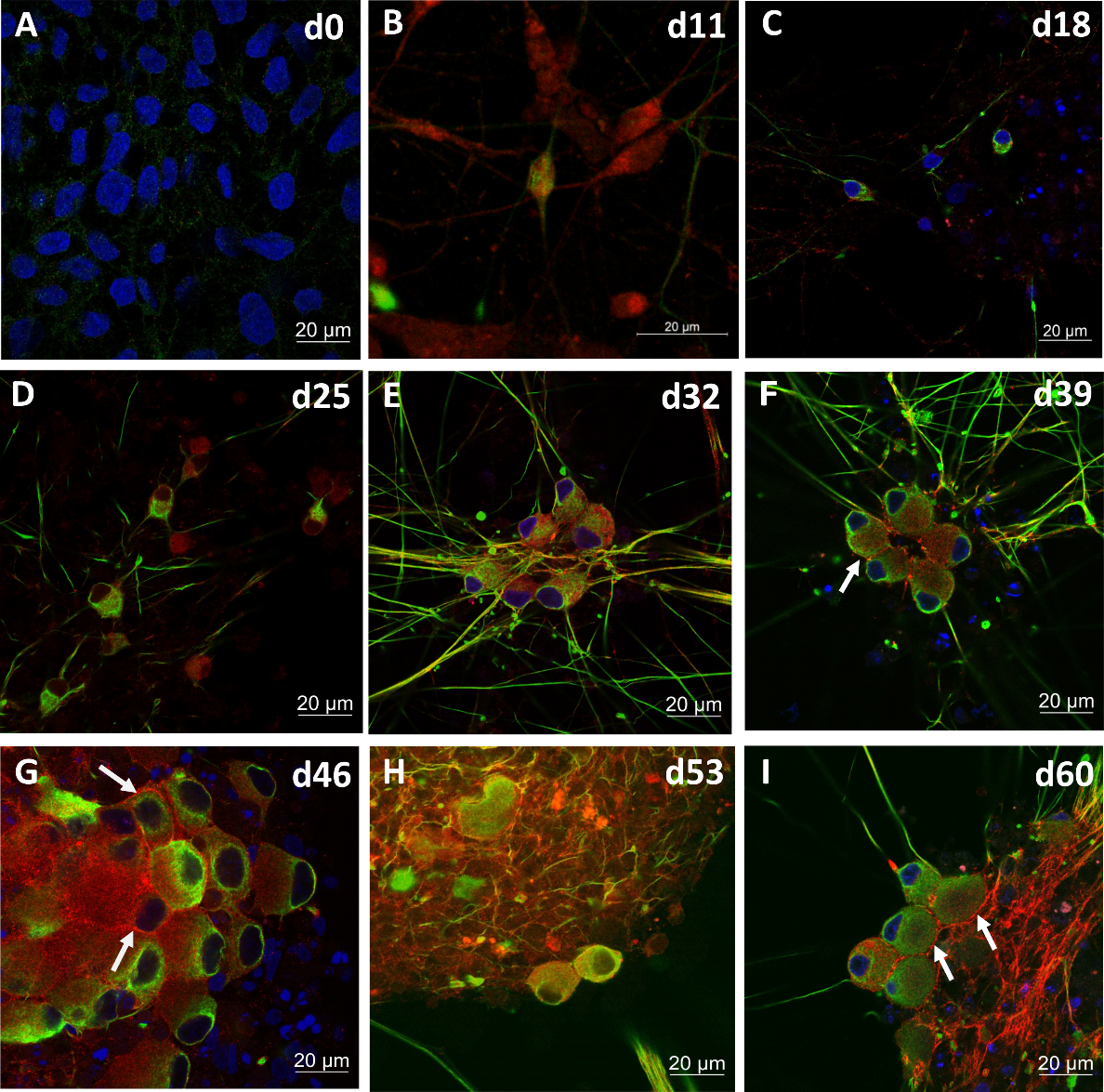


**Fig.S2** Confocal microscopy shows the localization and expression of HA-tagged Na_V_1.7 on iPSC-SNs in small molecule differentiation**. A-I. Double** immunolabelling (peripherin (green) and HA-tag (red)) of hiPSCs colonies at d0 of differentiation (**A**) to mature sensory neuron ganglia-like structures at d60 of differentiation using confocal imaging (**I**). The cell nuclei were stained in blue using DAPI (blue). The distinct surface expression of HA-tag is marked with white arrows. All the data represents an overlay of peripherin (green) and HA-tag (red) staining. (scale bar: 20µm)


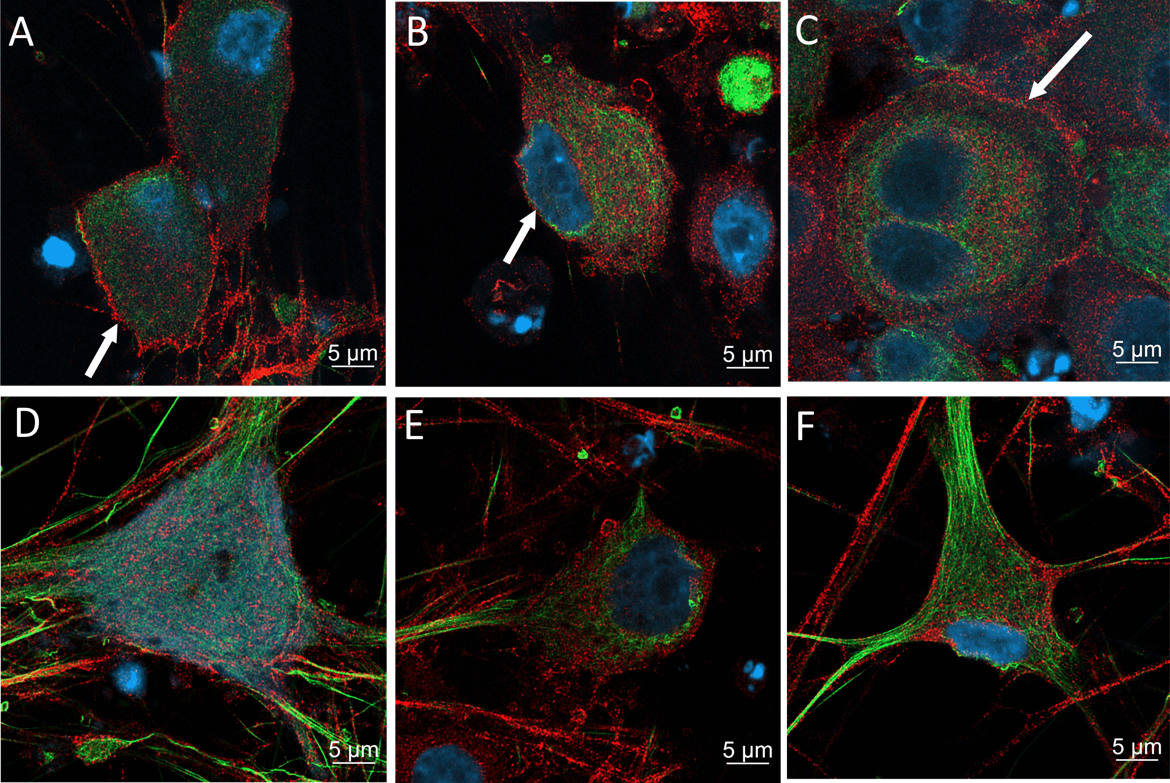


**Fig.S3** Localization of HA-tagged Na_V_1.7 on iPSC-SNs at d49 of NGN1 differentiation visualized by Airyscan microscope**. A-F**. double immunolabelled (peripherin (green) and HA-tag (red)) iPSC-SNs with or without clear membrane localization at d49 of differentiation using LSM 980 with Airyscan 2. White arrow heads showed clear membrane expressions of HA-tagged Na_V_1.7 in **A-C** which could not be found in **D-F**. The cell nuclei were stained in blue using DAPI (blue). The distinct surface expression of HA-Tag is marked with white arrows. Data represents an overlay of peripherin (green) and HA-tag (red) staining. (scale bar: 5µm)


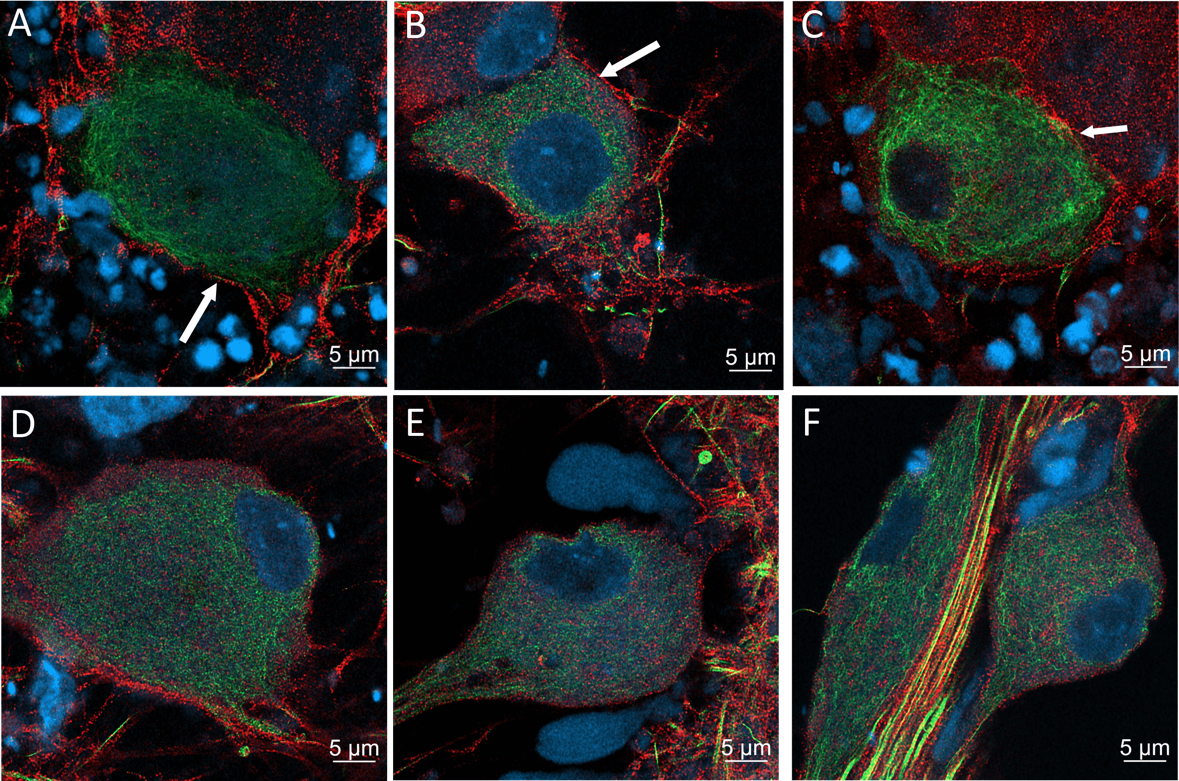


**Fig.S4** Localization of HA-tagged Na_V_1.7 on iPSC-SNs at d56 of NGN1 differentiation visualized by Airyscan microscope. **A-F**. double immunolabelled (peripherin (green) and HA-tag (red)) iPSC-SNs with or without clear membrane localization at d56 of differentiation using LSM 980 with Airyscan2. White arrow heads showed clear membrane expressions of HA-tagged Na_V_1.7 in **A-C** which could not be found in **D-F**. The cell nuclei were stained in blue using DAPI. The distinct surface expression of HA-tag is marked with white arrows. Data represents an overlay of peripherin (green) and HA-tag (red) staining. (scale bar: 5µm)


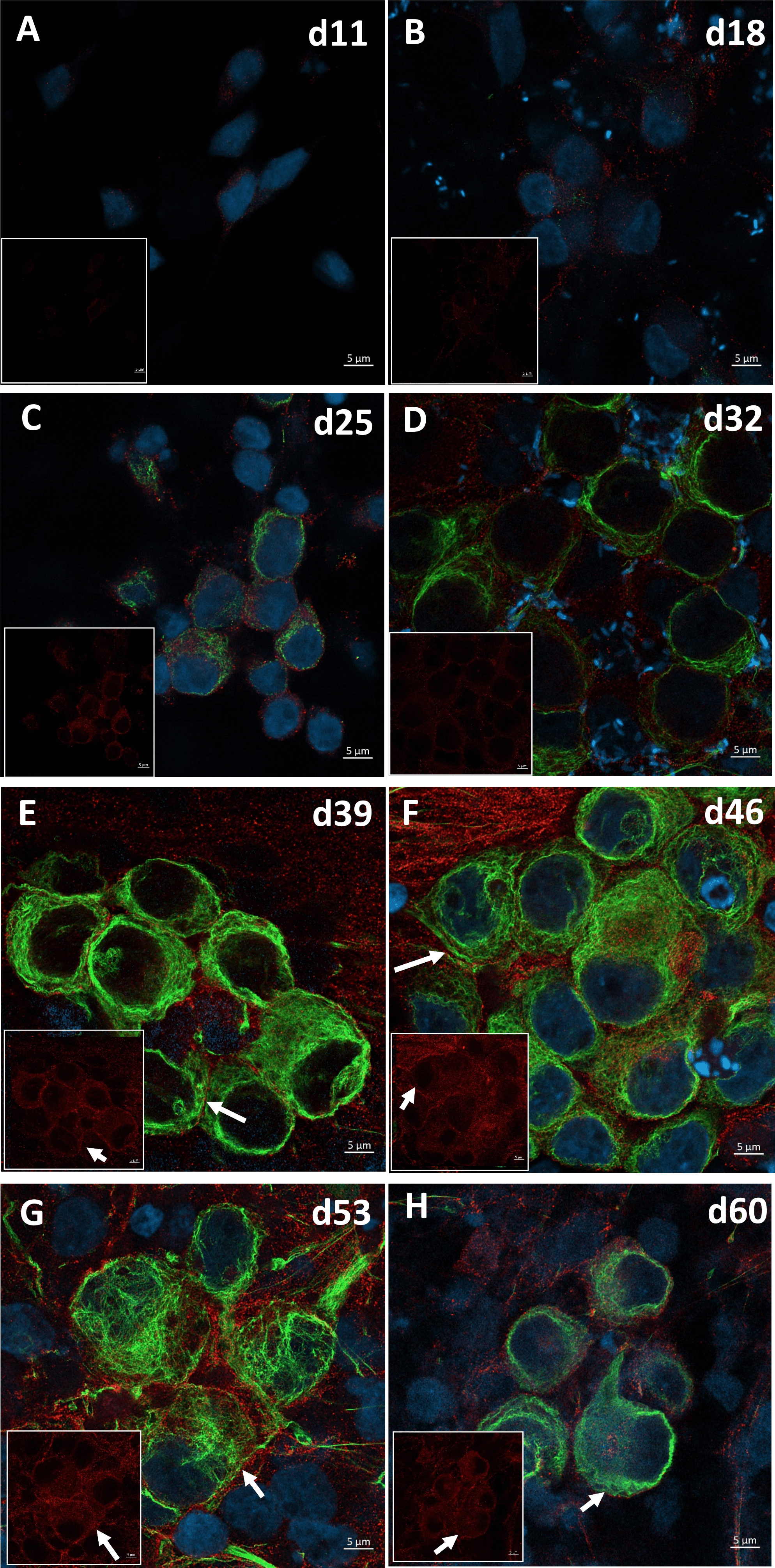


**Fig. S5** Na_V_1.7 protein surface expression start detectable from d39 during small molecule differentiation (Airyscan microscopy).**A-H**. double immunolabelled (peripherin (green) and HA-tag (red)) iPSC-SNs from d11 of differentiation (**A**) to mature sensory neurons at d60 of differentiation (**H**) using LSM 980 with Airyscan 2. The cell nuclei were stained in blue using DAPI (blue). The distinct surface expression of HA-tag is marked with white arrows. Data represents an overlay of peripherin (green) and HA-tag (red) staining. The pictures in white box showed representative picture of expression of HA-tagged Na_V_1.7 from each time point and a clear surface localization of Na_V_1.7 from d53-d60 of differentiation. White arrow shows the surface expression of HA-tagged Na_V_1.7 (scale bar: 5µm). The representative pictures are taken from two individual coverslips iPS-SNs derived from one small molecule differentiation and 4-7 areas per each timepoint have been examined in total.

Supplementary Videos

**Video 1 Z-stack video of** **localization of HA-tagged Na_V_1.7 on iPSC-SNs at d49 of NGN1 differentiation visualized by Airyscan microscope**

Data shows different focal distances of peripherin (green) and HA-tag (red) staining of iPSC-SNs at d49 of NGN1 differentiation using LSM 980 with Airyscan 2. The reliable detection of surface localization of HA-tagged Na_V_1.7 could be found at several stages. The cell nuclei were stained in blue using DAPI (blue). (scale bar: 5µm)

**Video 2 Z-stack video of** **localization of HA-tagged Na_V_1.7 on iPSC-SNs at d56 of NGN1 differentiation visualized by Airyscan microscope**

Data shows different focal distances of peripherin (green) and HA-tag (red) staining of iPSC-SNs at d56 of NGN1 differentiation using LSM 980 with Airyscan 2. The reliable detection of surface localization of HA-tagged Na_V_1.7 could be found at several stages. The cell nuclei were stained in blue using DAPI (blue). (scale bar: 5µm)

# Supplementary Tables

# **Table 1 List of primers used in this study**

| Primers | | Sequence (5' →3') |
| --- | --- | --- |
| HA-tag | Forward primer | CGT GCT TAT AGA CGT TAC CG |
|  | Reverse primer | AAT CTG GAA CAT CGT ATG GG |
|  | Flanking primer | AAC CAT CTG CTA ATG CTG CC |
| GAPDH | Forward primer | AGC CAC ATC GCT CAG ACA C |
|  | Reverse primer | GCC CAA TAC GAC CAA ATC C |
| Na_V_1.7 | Forward primer | CAC AAT CCC AGC CTC ACA GT |
|  | Reverse primer | CTG AGG AGC TTG ACC GGT TTA |
| HA-tag | Forward primer | CGT GCT TAT AGA CGT TAC CG |
|  | Reverse primer | AAT CTG GAA CAT CGT ATG GG |
| TUJ1 | Forward primer | CTC AGG GGC CTT TGG ACA TC |
|  | Reverse primer | CAG GCA GTC GCA GTT TTC AC |
| peripherin | Forward primer | ACA ACC TCG TGC TCT TCC G |
|  | Reverse primer | TGG CTC TCC ACA CTC ACC T |
| TRKA | Forward primer | CAG GAC TTC CAG CGT GAG G |
|  | Reverse primer | GCA GCT TGG CAT CAG GTC |
| TRPV1 | Forward primer | GCA CAG GAG AGC AAG AAC ATC |
|  | Reverse primer | GTC CAG TTC ACC TCG TCC AC |
| NGN1 | Forward primer | GCC TCC GAA GAC TTC ACC TAC C |
|  | Reverse primer | GGA AAG TAA CAG TGT CTA CAA AGG |
| SOX10 | Forward primer | ATG TCA GAT GGG AAC CCA GA |
|  | Reverse primer | GTC TTT GGG GTG GTT GGA G |

# **Table 2 List of primary antibodies and second antibodies used in this study**

| Target | Isotype | Dilution | Supplier | Catalogue number |
| --- | --- | --- | --- | --- |
| Stemlight™ Pluripotency transcription factor kit | Rabbit IgG | 1:200 | Cell Signaling technologies, USA | 9093 |
| Anti-human TRA-1-60 | Mouse IgM | 1ul/ 100ul for 10^5^ cells | Biolegend ,USA | 330610 |
| Anti-human SSEA-4 | Mouse IgG3 | 1ul/ 100ul for 10^5^ cells | Biolegend ,USA | 330408 |
| PE mouse IgM isotype control | Mouse IgM | 1ul/ 100ul for 10^5^ cells | Biolegend ,USA | 401609 |
| Mouse IgG3  isotype control | Mouse IgG3 | 0,5ul/ 100ul for 10^5^ cells | Biolegend ,USA | 401321 |
| HA-tag | Rabbit IgG | 1:800 | Cell Signaling technologies, USA | 3724S |
| peripherin | Mouse IgG2a | 1:500 | Santa-Cruz Biotechnology, USA | SC-377093 |
| βIII-tubulin | Chicken IgY | 1:500 | Novus Biologicals, USA | NB100-1612 |
| AF594 Goat Anti-rabbit IgG(H+L) | Goat IgG | 1:500 | Life technologies, USA | A-11037 |
| AF488 Goat Anti-mouse IgG (H+L) | Goat IgG | 1:500 | Life technologies, USA | A-11001 |
| AF555 Goat Anti-rabbit IgG(H+L) | Goat IgG | 1:500 | Life technologies, USA | A-21429 |
| AF633 Goat Anti-chicken IgY(H+L) | Goat IgG | 1:500 | Life technologies, USA | A-21103 |

# **Table 3 Sequence of the oligonucleotides used to generate HA-tagged NaV1.7 hiPSC lines**

For the donor template, the nucleotide in bold and underlined indicates the silent mutation introduced to disrupt the PAM site. The sequence highlighted in bold red indicates the HA-tag coding sequence and the novel stop codon is indicated in bold blue letter.

| Oligo name | 5´ --- 3´ sequence |
| --- | --- |
| gRNA_1 | AGACAAAGGGAAAGACAGCA |
| Donor template (144 nt) | A TAT GAA CAA GAC AGA ACA GAA AAG GAA GAC AAA GGG AAA GAC AGC AAA GAA AGC AAA AAA TAC CCA TAC GAT GTT CCA GAT TAC GCT TAG AGC TTC ATT TTT GAT ATA TTG TTT ACA GCC TGT GAA AGT GAT TTA TTT GTG TT |
| SCN9A_HA_FRW | CGTGCTTATAGACGTTACCG |
| SCN9A_HA_REV | AATCTGGAACATCGTATGGG |
| SCN9A_REV | AACCATCTGCTAATGCTGCC |
